# Perceiving the evil eye: Investigating hostile interpretation of ambiguous facial emotional expression in violent and non-violent offenders

**DOI:** 10.1371/journal.pone.0187080

**Published:** 2017-11-30

**Authors:** Niki C. Kuin, Erik D. M. Masthoff, Marcus R. Munafò, Ian S. Penton-Voak

**Affiliations:** 1 Penitentiary Institution Vught, Vught, the Netherlands; 2 School of Experimental Psychology at the University of Bristol, Bristol, United Kingdom; 3 MRC Integrative Epidemiology Unit at the University of Bristol, Bristol, United Kingdom; Leiden University, NETHERLANDS

## Abstract

Research into the causal and perpetuating factors influencing aggression has partly focused on the general tendency of aggression-prone individuals to infer hostile intent in others, even in ambiguous circumstances. This is referred to as the ‘hostile interpretation bias’. Whether this hostile interpretation bias also exists in basal information processing, such as perception of facial emotion, is not yet known, especially with respect to the perception of ambiguous expressions. In addition, little is known about how this potential bias in facial emotion perception is related to specific characteristics of aggression. In the present study, conducted in a penitentiary setting with detained male adults, we investigated if violent offenders (*n* = 71) show a stronger tendency to interpret ambiguous facial expressions on a computer task as angry rather than happy, compared to non-violent offenders (*n* = 14) and to a control group of healthy volunteers (*n* = 32). We also investigated if hostile perception of facial expressions is related to specific characteristics of aggression, such as proactive and reactive aggression. No clear statistical evidence was found that violent offenders perceived facial emotional expressions as more angry than non-violent offenders or healthy volunteers. A regression analysis in the violent offender group showed that only age and a self-report measure of hostility predicted outcome on the emotion perception task. Other traits, such as psychopathic traits, intelligence, attention and a tendency to jump to conclusions were not associated with interpretation of anger in facial emotional expressions. We discuss the possible impact of the study design and population studied on our results, as well as implications for future studies.

## Introduction

Many intrapersonal and contextual variables have been investigated in the search for risk factors for aggressive behavior. One line of investigation has focused on the general tendency of aggression-prone individuals to infer hostile intent in others, even in ambiguous circumstances. This is referred to as the ‘hostile attribution bias’ [1]. This bias may play an important role in perpetuating negative cognitive schemas, and contribute as a causal factor in negative social interactions (because: ‘when I expect you have hostile intentions towards me, I will react more defensively or offensively towards you’), potentially resulting in aggression and thereby enhancing the cycle of violence [2]. This tendency has mostly been investigated in children in study designs where participants were presented with a story describing an interpersonal situation with ambiguous cues, and asked to make assumptions about the motives of the story characters. In such study designs, aggression prone individuals have consistently shown to infer more hostile intent than normal controls [3], especially with respect to reactive aggression [4–8]. However, other relevant and more basic aspects of social information processing have not been intensively investigated in this context, even though there are indications that hostile interpretation of social signals occurs in very early stages of information processing [9]. This suggests the existence of a more basic hostile interpretation bias next to a hostile attribution bias. One potential factor to consider in this respect is facial emotion perception, since in social interactions, facial information processing is one of the most immediate sources of information [10]. Although a hostile interpretation bias in facial emotion perception could play an important role in aggression, this possibility has received little attention in empirical studies, especially with respect to ambiguous (instead of pronounced expressive) faces [10]. In addition, it may be argued that such ambiguous or neutral facial expressions may be more common in social interactions in daily life [11], making this research particularly relevant. To date, few studies have shown that the hostile interpretation bias in people with aggressive tendencies manifests itself indeed in the basic level of facial emotion perception [10, 12–14]. For example, Schönenberg and Jusyte [10] found that violent offenders rated morphed ambiguous faces on an happy-angry or fearful-angry dimension as more angry than healthy volunteers, while no clear differences between groups were present on the more extreme exemplars (e.g., non-ambiguous) of these continua [10]. This biased perception appeared to be restricted only to emotional dimensions with a component of anger. However, in a recent study among forensic outpatients from Smeijers, Rinck, Bulten, Van den Heuvel and Verkes [12] a highly generalized hostile interpretation bias was manifested in perception of emotionally ambiguous faces with respect to multiple emotions (not only anger, but also disgust and fear). Moreover, this study showed that this bias appeared to be characteristic of pathological aggression, regardless of intelligence or a psychiatric diagnosis of antisocial or borderline personality disorder or intermittent explosive disorder. Within the aggression spectrum, aspects of reactive aggression were associated (albeit inconsistently) with the hostile interpretation bias [12], in accordance with earlier studies on the hostile attribution bias [2, 4–8]. Interestingly, some promising studies have shown that interventions to decline hostile interpretation of facial expressions, may prove to be successful in reducing aggressive ideation and behavior [15–17], and may be associated to changes in activation in the lateral orbitofrontal cortex and amygdala in youth with disruptive mood dysregulation disorder [17].

Furthermore, suggestions have been made that additional factors are in play when it comes to the hostile interpretation bias in the perception of emotionally ambiguous faces. In the study of Smeijers et al. [12], for example, a link was found between conscious self-serving distortions and the more implicit hostile interpretation bias in facial emotion perception. In addition, Schönenberg and Jusyte [10] showed that the intensity of angry expressions was systematically overrated by the violent offender group, which may suggest that violent offenders felt more confident about the decisions they made than normal controls, who may have been more conscious of the ambiguous aspects of the emotional expressions. This could imply that the presence of a hostile interpretation bias may be related to a tendency to jump to conclusions. Another aspect to consider is the suggestion that subjects with psychopathy may have a more pronounced hostile interpretation bias than offenders without psychopathy [18, 19]. Although no evidence was found for a relation to antisocial personality disorder [12], the hypothesis that psychopathy mediates the relation between perceiving more hostile intent and aggression remains to be investigated [10].

Thus, although studies on hostile interpretation of ambiguous facial expressions in relation to aggression have emerged in recent years, questions remain about the specific nature of this relationship. This concerns, for example, differences between aggression typologies (reactive, impulsive aggression versus proactive, instrumental aggression), the role of psychopathic personality traits, and cognitive biases concerning, for example, confidence of judgment. A better understanding of these relationships could be helpful in the development and selection of treatment programs for aggression reduction. Therefore, the aim of the present study was to investigate the presence of a hostile interpretation bias in perception of emotionally ambiguous faces on a morphed sequence on a happy-angry dimension, in a group of detained male violent offenders in contrast to non-violent offenders and healthy volunteers. More specifically, we investigated associations between aggression subtypes, other characteristics of criminal (aggressive) behavior, and the hostile attribution bias toward ambiguous facial expressions. We also investigated if hostile interpretation is positively correlated with traits of psychopathy, measured through self-report with the Psychopathic Personality Inventory revised (PPI-R) [20], and a tendency to jump to conclusions, assessed with the beads-in-a-jar task [21].

## Materials and methods

### Setting and participants

Participants from offender groups were recruited from different regimes in a large prison setting in the Netherlands for adult male offenders (Penitentiary Institution Vught). Participants (*n* = 85) were suspects or, for the larger part, convicts of criminal acts, ranging from minor offenses to severe violent crimes. Sentences varied from several weeks to life-long imprisonment, sometimes in combination with special treatment programs. The healthy volunteer group (*n* = 32) consisted of male employees of the prison service, who volunteered for the study and who all had a clean criminal record.

**Fig 1:**
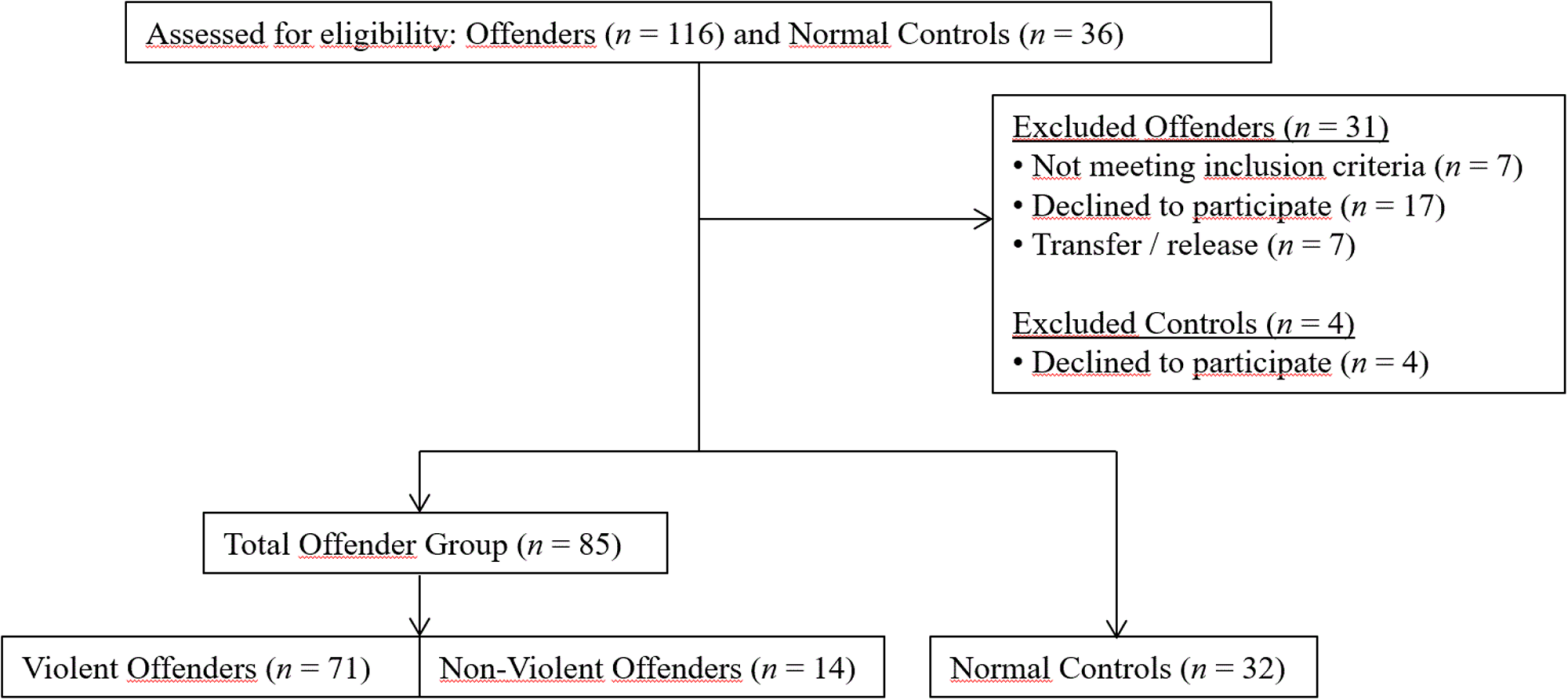
Flowchart of the inclusion process.

Participants were excluded from the study when they were known to be suffering from, or had suffered from during the last 6 months, an acute episode of a major psychiatric disorder prior to testing (e.g., major manic, depressive or psychotic episode) or had been previously diagnosed with an autism spectrum disorder. Additionally, participants were excluded if staff members expressed their concerns for the safety of the researcher (see Fig 1 for a flowchart of the inclusion process). Imprisoned participants were allocated to either the violent offender group (if they had a history with at least one conviction for a violent crime; *n* = 71) or the non-violent group if they had only been convicted for non-violent crimes; *n* = 14). Violent offenses included, for example, convictions for assault and battery, manslaughter, murder, sex offenses or arson with risk for persons. Non-violent offenses included convictions for drug crimes, fraud, serious traffic offenses or thievery. More detailed information on the study population and control groups can be found in Table 1. The groups were matched for gender (all participants were male), but were not individually matched with respect to age and intelligence.

**Table 1:** Descriptive data of the three study groups(violent offenders: VO, non-violent offenders: N-VO and normal controls: NC) and outcomes of post-hoc comparisons.

Measure	VO (*n* = 71) Mean (range, *sd)*	N-VO (*n* = 14) Mean (range, *sd)*	NC (*n* = 32) Mean (range, *sd)*	VO – N-VO (*p* – value)	Post-Hoc VO – NC (*p* – value)	N-VO – NC (*p* – value)
Age	36.56 (20–74, 11.76)	37.43 (18–60, 10.38)	41.75 (19–60, 11.17)	1	0.107	0.724
Educational level^1^:	3.10 (1–6, 1.10)	3.64 (2–5, 84)	4.16 (3–5,.52)	0.154	<.001	0.278
IQ (RSPM)	84.76 (70–124, 11.99)	85.61 (70–109, 11.90)	97.28 (79–124, 10.88)	1	<.001	0.007
TMT-A	33.38	26.68	25.63	0.212	0.013	1
TMT B/A	2.81	2.37	2.19	0.385	0.01	1
Beads task	5.21	3.43	6.13	0.724	1	0.32
PPI-r Total score	347.03	343.57		0.749		
Total number of convictions	24.93	10.36		0.023		
Number of previous violent convictions	4.96	0		<.001		
Number of previous non-violent convictions	19.03	9.43		0.092		
BPAQ-r total	27.15	22.93		0.163		
BPAQ-r physical aggression	7.75	6.29		0.188		
BPAQ-r verbal aggression	5.73	4.93		0.274		
BPAQ-r rage	6.2	5.29		0.328		
BPAQ-r hostility	7.46	6.43		0.297		
IPAS-30 total	73.54	65.61		0.219		
IPAS impulsive scale	24.51	22.5		0.431		
IPAS instrumental scale	19.89	17.9		0.348		
RPQ total	15.9	11.15		0.121		
RPQ reactive scale	9.82	8.08		0.266		
RPQ proactive scale	6.08	3.08		0.068		
SDAS^2^	3.78	5.38		0.378		

Note.

^1^ Educational level was based on the classification system of Verhage [22] in Dutch education with 6 levels of education: (1) not graduated from primary school, (2) only graduated from primary school, (3) vocational education, (4) Secondary vocational education, (5) Higher vocational education, (6) academic education.

^2^ ‘SDAS’ is based on mean ratings of weekly assessments by staff-members of behavioral observation during four weeks.

Abbreviations: *IQ*: Intelligence Quotient; *RSPM*: Raven Standard Progressive Matrices; *TMT*: Trail Making Test; *PPI-r*: Psychopathic Personality Inventory revised; *BPAQ-r*: Buss-Perry Aggression Questionnaire revised; *IPAS-30*: Impulsive/Premeditated Aggression Scales; *RPQ*: Reactive-Proactive Aggression Questionnaire; *SDAS*: Social Dysfunction and Aggression Scale.

The study was approved by the scientific department of the Dutch Ministry of Justice and Security with respect to procedural and ethical aspects. All participants signed for informed consent after receiving both verbal and written information about the study.

### Measures

#### Self-report questionnaires

Aspects of aggression were assessed with three self-report questionnaires on the one hand and objective data (justicial records and staff rated behavioral scales) on the other hand. The questionnaires were all internationally applied, well investigated questionnaires to assess (aspects of) aggression, that had been translated to the Dutch language in previous studies. First, the Reactive-Proactive Aggression Questionnaire (RPQ) [23] was applied, which is a self-report questionnaire to assess the amount of aggressive behavior one shows in general. Besides adding up item-scores to retrieve a total score, two factor scores can be extracted for proactive and reactive aggression. Results from a large Dutch validation study show good test-retest reliability and convergent, criterion and construct validity, as well as good support for its two-factor structure [24]. The second self-report instrument for aggression was derived from the Buss-Perry Aggression Questionnaire (BPAQ) [25]. In the present study, a revised version of this instrument (the BPAQ-r) was applied, since this shorter 12-item form showed better psychometric characteristics than the original BPAQ in a Dutch population of aggressive offenders [26]. The BPAQ-r has the same subscales as the original BPAQ: a total and four factor scores: ‘physical aggression’, ‘verbal aggression’, ‘rage’ and ‘hostility’. Finally, the Impulsive/Premeditated Aggression Scales (IPAS-30) [27] were administered. This 30-item self-report instrument provides a total aggression score as well as two factor scores for impulsive and premeditated aggression. It’s good psychometric properties and two-factor structure were confirmed in a previous study among Dutch prisoners, although the subscales were highly correlated [28].

Psychopathic personality traits were assessed through self-report with the Psychopathic Personality Inventory revised (PPI-R) [20]. This questionnaire was selected for the present study because of its ease to administer and because it is among the most frequently used self-report assessments for psychopathy [29]. Although this instrument provides detailed subscale scores on multiple levels, these factor scores appear to have inconsistent psychometric properties [29, 30]. Therefore, in the present study only the score on the total psychopathy index was included as a measure of the total amount of psychopathic traits.

#### Behavioral observations

Objective data on aggression came from structured behavioral observation with the Social Dysfunction and Aggression Scale (SDAS-11) [31]. The inter-observer reliability was moderate and the convergent validity was good in a previous Dutch study [32]. This observational instrument is intended to score behavior during the past week, which leaves too great a margin for random fluctuations in behavior for the purposes of the present study. Therefore, the SDAS was rated over four weeks to obtain a robust baseline measure of aggression. Staff members were instructed to score the SDAS-11 four times, with an interval of one week at a time. Behavior of the participants was rated on different aspects of aggression (for example verbal or physical aggression) for each week. Mean total scores from these four trials were calculated for the present study’s purposes.

In addition, criminal records were searched to obtain information about the crime history (based on prior convictions) and the current crime. Not every crime results in a conviction and conviction rates may therefore give an underrepresentation of the actual total amount of committed crimes. However, using self-report data on criminal behavior instead, might lead to an even larger under-report [33].

#### Neuropsychological assessments

In addition to measures for aggression, neuropsychological assessments of emotion perception, intelligence, divided attention, and drawing conclusions were conducted.

**Fig 2:**
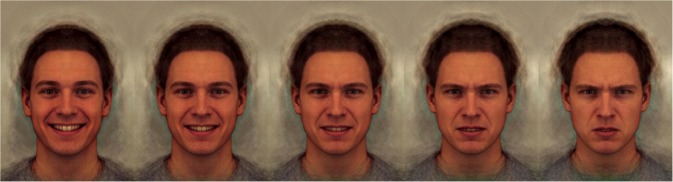
Pictures from the emotion perception task on a continuum from unambiguously happy to unambiguously angry with a neutral face in the middle of the spectrum.

The emotion perception task that was administered in the present study was developed by Penton-Voak et al. [16] (see Fig 2). Prototypical happy and angry composite images were derived from 20 individual male faces showing a happy facial expression and the same 20 faces showing an angry expression. The original images came from the Karolinska Directed Emotional Faces [34]. These prototypical images were used as endpoints to generate a linear morph sequence that consists of fifteen images that change incrementally from unambiguously happy to unambiguously angry, with emotionally ambiguous images in the middle. Each of the fifteen steps on the continuum was presented three times so that, in total, participants completed 45 trials. Participants were instructed to rate these images as happy or angry, in a two-alternative forced-choice procedure administered by a computerised test in E-prime 2.0.

First a fixation cross appeared (for 1500–2500 milliseconds, randomly jittered), followed by a short presentation of one of the faces on the happy-angry continuum (for 150 milliseconds) followed by a mask of visual noise (for 150 milliseconds), at which point participants rated the face as either happy (by pressing ‘C’) or angry (by pressing ‘M’). Within participants, classification responses to morph continua tend to shift monotonically from one expression response to the other across the continuum presented. Therefore, a simple estimate of the balance point, the ‘threshold-score’ (which represents the point at which a participant is equally like to respond happy or angry) can be derived by counting the number of happy responses as a proportion of the total number of trials [16]. The emotion perception task is scored so that lower scores reflect a tendency to rate the faces as angry, while higher scores reflect a tendency to rate the faces as happy.

To assess general intelligence, the Raven Standard Progressive Matrices (RSPM) were administered, a non-verbal intelligence test [35] in which abstract reasoning is essential. Participants are instructed to fill in missing parts in a pattern, choosing from a set of options. Scores on the RSPM correlate moderately to highly with IQ-scores on general intelligence tests [36]. Although this makes the instrument less suitable to assess general intelligence for individual diagnostic purposes, it was selected for the present study because it provides a good estimation of intelligence, has a relatively short completion time and, most importantly, is applicable for not native Dutch speakers. Dutch norms were applied to calculate percentile scores [36], which were subsequently converted into IQ-estimates.

Cognitive flexibility and divided attention were assessed with the Trail Making Test (TMT) [37]. It requires speed and good visual scanning skills, as well as inhibitory control and visual/spatial sequencing [36, 38]. In condition A, participants are instructed to draw lines between numbers as fast as possible (1-2-3-4-…), while in condition B, the instruction is to draw lines between numbers and letters and switch between those (1-A-2-B-3-C-…). The time to complete these tasks is regarded as a good indicator for processing speed (condition A) and cognitive flexibility and divided attention (condition B in contrast to condition A).

The beads-in-a-jar task [21] is a well-known paradigm to assess a tendency to draw conclusions after little information. Participants are shown two virtual jars filled with beads on a computer screen and are told that one jar contains 80 orange and 20 black beads, while the other jar contains exactly the opposite. One by one beads are drawn from one of the two jars. After each drawing participants are asked if they know to which jar the beads belonged or if they want to await a next draw. The test score depends on the number of drawings of beads, before reaching a conclusion (lower scores reflect a stronger tendency to jump to conclusions). This test is mostly known from studies with delusional patients, but appears to be specifically related to an analytic cognitive style (the willingness or disposition to critically evaluate outputs from intuitive processing and engage in effortful analytic processing) [39].

### Procedure

Participation in the study was voluntary. Research assistants recruited detained participants in the institution through posters and information letters, as well as through personal contact. Potential participants in the normal control group were approached by e-mail.

The offender groups completed both neuropsychological testing and measures of aggression. Healthy volunteers only completed neuropsychological testing during one session. For each participant within the offender groups, the study procedure lasted for four weeks in total, to be able to complete four SDAS-11 registrations. Participants usually had three meetings with a research assistant. In the first meeting specifics of the study were repeated. After signing informed consent, general personal data were gathered and the RSPM was assessed. During the second meeting the remaining testing took place following a structured protocol, which was part of a larger study. Feedback on individual test performance was offered to all participants. This was subsequently conveyed and explained to each participant, who wished so, in the last meeting.

### Statistical procedure

Data were analyzed using IBM SPSS Statistics^®^ software, version 22. Before conducting any statistical analyses, both within the total sample and within subgroups, all variable distributions were inspected for their approximate fit to a normal distribution, linearity, homoscedasticity, and homogeneity of variance. Variables within large groups were inspected visually, using histograms and scatter-, P-P, and Q-Q plots. For small subgroups, model-fit for a normal distribution was calculated using Kolmogorov-Smirnov test. Due to unequal group sizes, Levene’s test was performed to verify the homogeneity of variance across the three samples. In case of normal distributions, Levene’s test was based on mean scores, while in case of non-normal distributions this was based on the median.

Descriptive statistics for the three groups (violent offenders, non-violent offenders and healthy volunteers) were calculated and compared by means of a one-way ANOVA with Bonferroni corrected post hoc tests. If the dependent variable (threshold scores) correlated significantly with potentially confounding variables, such as age, intelligence, and measures of attention (speed on TMT-A and TMT-A/B), and if subgroups were not matched on these variables, these confounding variables would subsequently be included as covariates in any further statistical analyses. Both parametric and non-parametric two-tailed bivariate correlations were applied here. For comparisons between both offender groups, independent sample t-tests were conducted.

A oneway ANOVA with Bonferroni corrected post hoc tests were conducted to determine if the three groups, violent offenders (*n* = 71), non-violent offenders (*n* = 14) and normal controls (*n* = 32), differed on their threshold scores.

Finally, a multiple regression analysis with forced entry was conducted within the group of violent offenders to predict threshold scores from variables of aggression. Variables were entered individually. Since there were too many aggression variables to include altogether in the regression analysis, only variables were included that correlated significantly to threshold scores of the emotion perception task or approached significance. Two-tailed bivariate correlations were calculated (either Spearman’s *ρ* or Pearson’s *r*, depending on the distribution of the data).

To be able to determine the detectable effect size given the recruited sample size, a sensitivity power calculation was conducted using G*power. Given the sample size of the smallest group (14), an effect size of *f* = .05 should be detectable with 80% power. That is equivalent to slightly more than half a standard deviation difference between each group across the three groups.

## Results

### Descriptive data

Threshold scores in all subgroups showed approximately normal distributions, and measures of skewness and kurtosis were all within acceptable limits. However, many of the other variables were clearly not normally distributed. The assumptions of linearity and homoscedasticity of variance were confirmed for all variables within each of the three groups.

As can be seen in Table 1, the three groups differed significantly in level of education, processing speed on TMT-A, and processing speed on TMT-B corrected for speed on TMT-A. Post hoc tests revealed that these results could mostly be attributed to lower scores on these measures for violent offenders in contrast to healthy volunteers. There was no statistical evidence that intelligence scores differed between offender groups, *M*_violent offenders_ = 84.76, *M*_non-violent offenders_ = 85.61, *p* = 1.000. However, both these groups scored significantly lower on the RSPM than the normal controls, *M*_normal controls_ = 97.28, *p* <.001, *p* = .007 respectively. Of these variables, there was only evidence that age correlated with threshold scores, although exclusively within the violent offender group, *ρ* = .318, *p* = .007. Since the three groups did not differ substantially in age and beads task scores, it was decided to not include these variables as a covariate in group equations for threshold scores, but they were included in the regression analysis.

Violent and non-violent offenders differed significantly on their delinquent histories only. Interestingly, violent offenders were convicted for more crimes in total, compared to their non-violent counterparts, including more non-violent crimes than the non-violent group (although the latter difference was not significant). In addition, mean scores of the violent offenders on all of the other measures of aggression (both self-report and staff-rated) were approximately equal to mean scores of their non-violent counterparts.

### Performance on the emotion perception task

**Fig 3:**
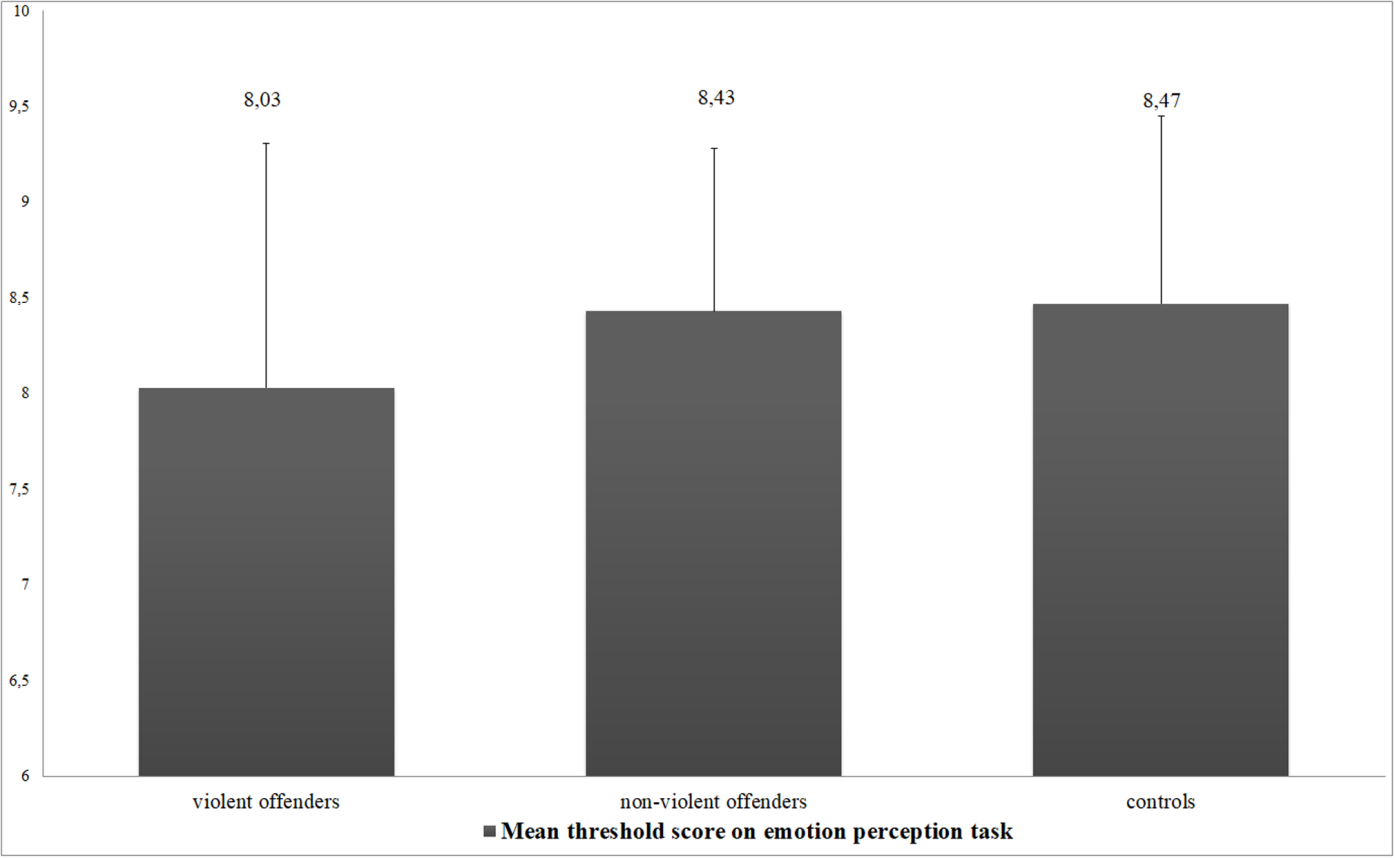
Average threshold scores on the emotion perception task of the three groups. Error bars represent standard deviations.

The oneway ANOVA to compare threshold scores on the emotion perception task between the three groups revealed no clear evidence of group differences (*F*(2, 114) = 1.910, *p* = .153), as did post-hoc tests. Mean scores and standard deviations of all groups are displayed in Fig 3.

**Fig 4:**
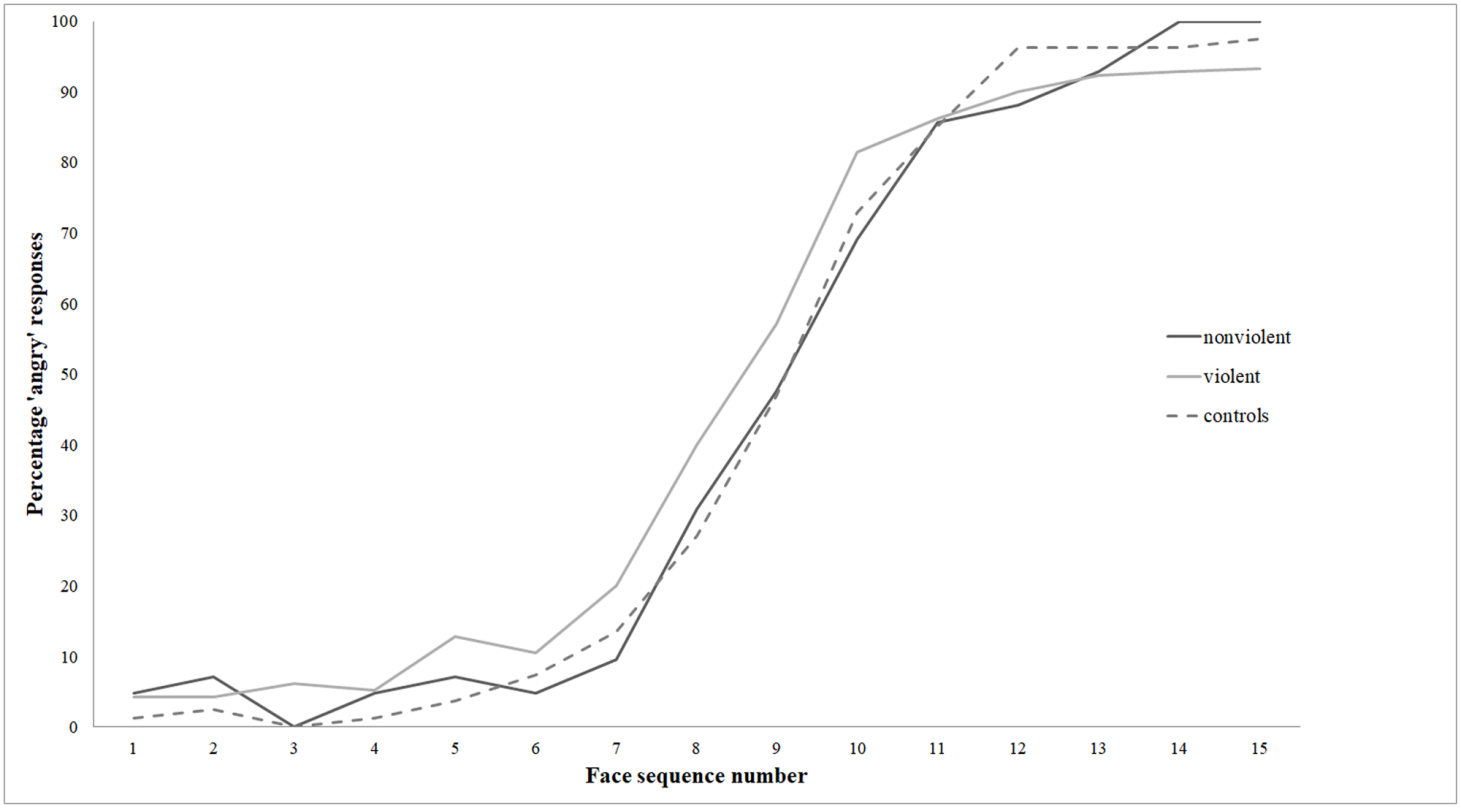
Percentage of ‘angry’ responses for each of the fifteen images on the happy-angry continuum for violent offenders, non-violent offenders and normal controls.

As can be seen in Fig 4 the curves of the mean percentages of ‘angry’ responses across all fifteen faces of the happy-angry continuum are approximately similar for the three groups. Nevertheless, there was a tendency for violent offenders to rate faces as slightly more angry than the other groups, at least in the ambiguous middle-section of the happy-angry continuum.

Because the variance of the threshold scores appears to be larger in the violent offender group than in the other groups (see Fig 3), it was decided to investigate if it was possible to explain part of this variance by scores on the other neuropsychological measures and aggression variables. Within the violent offender group (*n* = 71), threshold scores only correlated weakly with age (*r* = .30, *p* = .011), beads task score (*ρ* = -.20, *p* = .097), BPAQ-r total score (*ρ* = -.23, *p* = .050) and BPAQ-r rage (*ρ* = -.20, *p* = .097) and hostility scores (*ρ* = -.23, *p* = .056). However, there was no clear evidence that threshold scores correlated to any of the other self-report or observational measures on aggression, history of delinquency, PPI-r total score, scores on the RSPM or TMT in the violent offender group. These variables were therefore not included in the regression analysis.

Table 2 displays the outcome of the regression analysis. There was only evidence that age and the BPAQ-r subscale ‘hostility’ contributed to the prediction of threshold scores in the final step. Adding these variables to the model increased the explained variance of the threshold scores on the emotion perception task with approximately nine and seven percent respectively. In all steps, except for step 3, threshold scores were predicted better by the model than by the mean. There was no colinearity between BPAQ-r subscales and total score.

**Table 2:** Final results of the regression analysis, displaying linear model predictors (in order of entry in the regression analysis) of threshold scores on the emotion perception task in the violent offender group (*n* = 71).

	*B* (95% confidence interval)	*SE*	*β*	*p* *(α* = .*05)*
Step 5				
Constant	7.01 (5.50–8.62)	.78		*p* < .001
Age	.04 (.01 - .07)	.01	.39	*p* = .004
Beads task score	-.02 (-.08 - .04)	.03	-.09	*p* = .456
BPAQ-r rage score	-.07 (-.30 - .15)	.11	-.18	*p* = .527
BPAQ-r hostility score	-.16 (-.29 - -.03)	.06	-.42	*p* = .017
BPAQ-r total score	.04 (-.04 - .13)	.04	.34	*p* = .308

*Note*. Δ*R*^*2*^ = .013 for Step 5 (*ps* = .308). Prediction with this model versus prediction based on the mean was significant, *F*(5, 65) = 2.98, *p* = .017.

## Discussion

The first aim of the present study was to compare violent offenders to non-violent offenders and healthy volunteers with respect to their tendency to interpret ambiguous facial expressions as either angry or happy. Comparison of the average scores on the emotion perception task between the violent offenders, non-violent offenders and normal controls showed a difference in the expected direction, with violent offenders rating faces as slightly more angry than the other two groups. However, there was no clear statistical evidence to suggest that this difference was meaningful. A power calculation indicated that the achieved sample sizes in the present study were too small to be able to detect plausible group differences on the emotion perception task. The fact that the study was underpowered and that there were unequal groups sizes makes it difficult to interpret the results of our statistical tests. Interestingly, when looking to the data in a more qualitative approach, a similar trend was visible in the curves showing the percentage of angry responses on the emotion perception task: especially in the ambiguous faces, the curves showed a slight but consistent tendency for violent offenders to rate these faces as more angry than the non-violent offenders or normal controls. These findings are consistent with earlier findings where the same task was applied in juveniles [16], resulting in a similar curve. Even though these small trends were present, our findings are evidently less pronounced than those of three recent studies [10, 12, 40] in which a clear tendency for hostile perception of facial expressions was found within aggressive or forensic psychiatric groups in comparison to healthy volunteers or non-aggressive forensic controls. In fact, in one of these studies, this hostile perception was not limited to the angry spectrum, but manifested itself in perception of all six basic emotions [41]. Also, in a review of 15 studies on this topic, most of these studies revealed some sort of anger biased perception pattern with regard to the perception of facial expressions, albeit convincing consistency between these studies was lacking [2]. In line with our findings, several studies also found no clear statistical evidence for the presence of a hostile interpretation bias in relation to facial affect recognition [42–46]. Jusyte and Schonenberg [42], for example, found that violent offenders perform equal to normal controls in the detection of emotional versus neutral faces, but subsequently make errors categorizing these emotional cues to specific emotions. Violent offenders show such categorization deficiencies in fearful blends, but no support was found for a heightened sensitivity for anger. These authors therefore argued that aggression is not as much related to deficits in detection stages of social information processing, but is tied to categorization difficulties [42], and hence further distinctions should be made in specific stages of social cognitive processing [47].

Differences between study results may be due to diversity in study samples and approaches [2]. For example, two of the studies that showed no evidence for anger biases in perception of facial expressions were performed with either girls or women [43, 44]. In another study an association was observed between hostility and the perception of facial expressions of others as more angry, although only in men and not in women [12, 41]. However, the opposite has also been observed (i.e., a biased perception was found in women, but not in men) [48]. Facial affect processing has been shown to proceed differently in men and women at the level of the brain circuitry involved [49], which makes comparisons difficult. Another potential explanation for discrepancies between study results may be found in differences in task instructions. In two prior studies showing strong evidence for hostile interpretation bias [10, 41], participants were instructed to rate the likelihood or intensity of the perceived emotion in the portrayed face on a Likert scale, while in the present study only two opposite alternatives were provided (happy/angry). Adding a Likert scale to rate the intensity of the emotions could have contributed in more detailed variability of responses.

A second aim in our study was to gain more insight in the role of specific characteristics of aggression in relation to the hostile interpretation bias. One interesting finding in the present study was that the variance on the emotion perception task threshold scores was larger in the violent offender group than in the other two groups. This could be the result of potential heterogeneity of the violent offender group, and suggests that there may be individual differences, for example in specific components of aggression with subsequent different relations to hostile interpretation of faces. It was expected, for example, that impulsive/reactive aggression would be mostly correlated to hostile interpretation of faces, that more aggressive delinquent behavior would also be related to a stronger bias in emotion perception, and that psychopathic personality characteristics might explain part of the variance in the hostile perception of faces in violent offenders. Results of the regression analysis showed that in this respect only the hostility score of the BPAQ-r could predict outcome on the emotion perception task. Interestingly, the BPAQ-r hostility scale reflects the tendency to perceive hostility in the surroundings, and may therefore be logically most closely related to a hostile interpretation bias. This measure represents an indirect tendency for aggressive behavior, in contrast to the more pronounced measures for impulsive/reactive or instrumental/proactive aggression that were included in the study. However, these more explicit aspects of aggression were not strongly correlated with emotion perception scores in the violent offenders. In the recent study of Smeijers et al. [12], on the other hand, reactive aggression was significantly related to a hostile interpretation bias in facial emotion perception. However, this relationship was only present with respect to perception of fear and disgust, and not regarding happy or angry faces, just as in the present study. In addition, the data in the violent offender group show no consistent correlation between scores on the emotion perception task and psychopathic personality traits or any of the data from criminal records, such as the number of previously committed (violent) crimes. In line with these findings, Mellentin et al. [2] found in their review no evidence of a relation between psychopathic traits and anger biases in facial emotion perception.

These findings altogether suggest that a tendency to perceive negative, angry affect in facial expressions in violent offenders is associated with a general tendency to experience hostility, but does not translate in a clear relation with other aggressive or psychopathic personality characteristics.

In addition, the heterogeneity of the violent offender sample in the present study was also apparent in their criminal behavior patterns. For example, the sample consisted of first- and repeat- offenders, and mild to severely violent offenders. It could be argued that committing a violent crime by itself may not automatically signify a general aggressive tendency. This assumption is supported by our data, showing no clear differences between violent and non-violent offenders in their scores on aggression questionnaires. Another aspect to consider is that the violent offender group included sex offenders. Previous studies have shown that sex offenders (especially with minor victims) show distinct problems in emotion perception and attribution when compared to violent offenders [40, 50]. In the present study, however, sex offenders with minor victims showed similar threshold scores as other offenders in the violent offender group.

Other variables were assessed as well to control for potential disturbing influences. Intelligence and aspects of attention were not related to threshold scores of the emotion perception task in the violent offenders, and neither was the score on a task to assess a tendency to jump to conclusions. This suggests that hostile interpretation of faces is not related to low intelligence, problems with processing speed, problems with cognitive flexibility and divided attention, or overconfidence in decision making. However, we did find evidence for a correlation between age and perception of hostility in facial expressions in the violent offender group: the older the offenders, the less they rated the facial expressions as hostile. Whether this ‘age-effect’ was in fact linked to the number of years spent in prison has unfortunately not been investigated in the present study. This finding that older participants were less likely to (over)rate the faces as angry might explain why studies in aggressive children show pronounced hostile interpretation biases [51–53]. Although this finding may only represent differences in age cohorts, it may also give some reason for optimism: could time restore the hostile interpretation bias? Some support for this hypothesis may be found in literature on aging and emotion perception in general. Results from a meta-analysis on this topic indicate that older adults have increased difficulty recognizing basic emotions, including anger [54]. One suggested explanation for this phenomenon is that older adults shift attention towards more positive rather than negative cues, a ‘positivity bias’. Such a positivity bias coming with age might explain the found age-effect in the present study, but evidence for this is questionable. Although this theory was supported in some studies [55, 56], older adults appear to show an equally diminished ability to perceive happy facial expressions [54]. A better explanation for the decline in accuracy of emotion perception with age may be found in age-related neuropsychological changes in the social brain, more specifically change in the frontal and temporal lobes and/or changes in neurotransmitters [54]. How such neuropsychological and neuro-anatomical changes may relate to a potential decline in hostility biases of aggressive individuals would be something to consider in future studies.

Another important aspect to discuss with regard to the present study is the selection of the control group, since it may be questioned if the healthy volunteer sample of prison workers is representative for the general population. Prison workers may be hyper-vigilant to aggressive cues due to the nature of their job. In earlier studies on characteristics of Dutch prison workers, a positive correlation was found between exposure to violence and experiencing heightened aggression levels themselves, which was also related to type D personality [57, 58]. This could imply that the normal control group may also suffer from higher than normal levels of aggression and even a similar hostile interpretation bias. This was not corrected for in the present study. However, psychosocial risks for prison staff members have been shown to be highest for prison workers with direct contact to prisoners [59], while not all participants in the control group had frequent contact with inmates, since they had variable functions within the penitentiary institution. On the other hand, it may be argued that prison staff members represent an even better control group than healthy, naïve controls, since prison workers find themselves in partly similar conditions to those of prisoners for a large amount of time, and are still healthy individuals. This may make them even more comparable than controls, who remain in quite different environmental conditions. Furthermore, in other forensic studies with neuropsychological measures, control groups also consisted of staff members [60–63].

Aspects to consider in future studies are the experience of other types of negative affect and gender. The experience of negative affect, such as depression, might be important, because it has been shown that a relation between perceiving hostile intent and being aggressive towards others might manifest itself even more strongly in people with depressive symptoms [64]. Future studies should also incorporate measures for detection of body signals of emotion instead of only facial expressions. In one earlier study it has indeed been shown that the hostile interpretation bias also manifested itself in perception of body signals [65]. Furthermore, task paradigms should be altered to be able to target more specifically into specific social information processing stages [42].

Finally, the question arises if it is possible to restore a hostile interpretation bias in offenders, and if this would lead to a decline in hostility and anger/aggression. Results of three early studies give rise to optimism in this respect [15–17], which adds to the importance for future studies to not only explore, but also intervene. The present study has added to the knowledge of important aspects to consider in such investigations.

## Conclusions

The results of the present study partially support earlier findings, although these results are less pronounced. This is probably partly due to the fact that the study was underpowered and may also be related to differences in characteristics of task instructions. Contrary to expectations, no clear relations were found between hostile interpretation of facial affect and most of the measures concerning specific characteristics of aggression in violent offenders, which has not yet been assessed in this much detail in earlier studies. Only self-reported hostility was related to hostile interpretation of faces. In addition, emotion perception was not related to measures of criminality, psychopathy or a tendency to jump to conclusions. The findings of the present study could fuel the design of future studies on the topic, especially with respect to the need and capability of intervention studies on this level.
